# Supplementary material for: HIVprotI: an integrated web based platform for prediction and design of HIV proteins inhibitors
Source: J Cheminform. 2018 Mar 9;10:12. doi: 10.1186/s13321-018-0266-y (PMC5845081; doi:10.1186/s13321-018-0266-y)
Supplement: Supplementary file 2 — Additional file 2. Source code of HIVProtI web server. [file 13321_2018_266_MOESM2_ESM.zip › HIVprotI_Source-code/hivprotI/jsme/doc.html]

Installation of the JSME molecule editor


# Installation of the JSME molecule editor

## Introduction

This document describes how to embed JSME into a web page.  
The first section describes how to migrate pages that use the JME Java applet.  
The second part shows how to install JSME using JavaScript.

## Migration from JME to JSME

The HTML pages that use the "applet" tag can easily adapted thanks to the provided JavaScript code. Pages that use other methods to embed a Java applet,
using the "object" or the "embed" tags are presently not supported.

### HTML modification for pages using the "applet" tags

Many websites still use the JME Java applet. Migrating an HTML page
from JME to JSME is basically a two steps process:
  

- Include the JSME javascript code
- Rename the java APPLET tag to DIV tag

Example of simple html page with a Java JME applet:
  

<html>  
<head>  
<title>JME Example</title>  
</head>  
  
<body>  
<**applet** code="JME.class" name="JME" archive="JME.jar" width="360" height="315" id="JME">  
You have to enable Java and JavaScript in your browser to use JME!  
</**applet**>  
</body>  
</html>

  
The same page after migration to JSME:

<html>  
<head>  
**<script type="text/javascript" language="javascript" src="jsme/jsme.nocache.js"></script>**  
<title>JME Example</title>  
</head>  
  
<body>  
<**div** code="JME.class" name="JME" archive="JME.jar" width="360" height="315" id="JME">  
You have to enable JavaScript in your browser to use JSME!
</**div**>  
</body>  
</html>

  
  
This example page can be tested in your browser: JME\_to\_JSME\_simple.html
  

### Important warning

Due to an Internet Explorer bug, the id of any DIV element may never be "JSME".
  
  
Here is an example for which some older versions of Internet Explorer will fail:  

<**div** id="JSME" "code="JME.class" name="JME" archive="JME.jar" width="360" height="315" id="JME">  
You have to enable JavaScript on your machine !
</**div**>

### Accessing the applet variable in the JavaScript code

Java applets can be accessed in the JavaScript code in three different ways:  

- document.*JME* using a variable name
- document.*applets[0]* using the applet array
- document.getElementById("JME")> using the applet id

  
With JSME, the equivalent JavaScript applet can be accessed in the JavaScript code in two different ways:  

- document.*JME* using a variable name (identical to Java applets)
- document.*jsapplets[0]* using the jsapplets array (different to Java applets)

  
The third method is not supported.
  
  
If your HTML page uses the document.applets to access the JME applets, then all occurences of **document.applets** will need to be replaced by **document.jsapplets**.
  
  
  
Example:  

<div code="JME.class" **name="JME1"** archive="JME.jar" width="360" height="315" id="JME">  
You have to enable JavaScript on your machine !
</div>  
<div code="JME.class" **name="JME2"** archive="JME.jar" width="360" height="315" id="JME">  
You have to enable JavaScript on your machine !
</div>>

  
The code to access them using the variable name and the jsapplets array is shown below:

<input type="button" value="Get smiles via document.JME1" onclick="alert(**document.JME1**.smiles());">  
<input type="button" value="Get smiles via document.jsapplets[1]" onclick="alert(**document.jsapplets[1]**.smiles());">

  

### Timing issue with the initialization of the JavaScript applet using body.onload()

Java applets can be initialized using some JavaScript code after the page has finished loading. Unfortunately, the "body.onload()" method
that is usually used for this purpose cannot be used with JSME because some JSME instances might not be ready when the body.onload method is called.  
To solve this problem, the JSME initialization can call a function named "jsmeOnLoad" that contains custom initialization code.  
  
Example of simple html code with a "body.onload()" for the Java applet JME:  

<body onLoad="**loadJmeFile();loadMolFile();**">

  
Example of simple html code with an empty "body.onload()" and a "jsmeOnLoad" function for JSME:  

<script>  
function jsmeOnLoad() {  
    **loadJmeFile();loadMolFile();**  
}  
</script>  
  
<body onLoad="">

### Example of JME to JSME pages

- Simplest page
- Page showing how to invoke JSME and and to setup options
- The JME example 1 page adapted to JSME
- The JME example 2 page adapted to JSME
- The JME example 3 page adapted to JSME
- The JME example 4 page adapted to JSME

### Implementation note

After being loaded, the JSME JavaScript code scans the HTML page looking for all DIV elements have a code attribute as code="JME.class".
The JavaScript code that performs this task is available as a function named replaceAllAppletsByJSME().
For more information about this function, see the API section.

## Installation of JSME using JavaScript code

To install JSME in a web page requires three items:

- include the JSME JavaScript code
- define a function named jsmeOnLoad that will create a JSME instance
- define a HTML element where the created jsme will be placed

<html>  
  
<head>  
 **<script type="text/javascript" language="javascript" src="jsme/jsme.nocache.js"></script>** 
  
 **<script>  
 //this function will be called after the JavaScriptApplet code has been loaded.  
    function jsmeOnLoad() {  
        jsmeApplet = new JSApplet.JSME("jsme\_container", "380px", "340px");  
   }  
</script>** 
</head>  
<body>  
**<div id="jsme\_container"></div>**  
  
</body>  
</html>

The HTML code above can be tested here.  
  
The use of a the "jsmeOnLoad" function is necessary only if a JSME instance needs to be available immediately after the HTML page has been loaded. For more information, see the timing issue defined above.
  
  
The example code above is minimalist, it is actually not very useful because it does not show how to use the JavaScript variable "jsmeApplet" that was created.  
Here is a new example with a button that displays the SMILES of the molecule drawn in the editor:  

<html>  
  
<head>  
<script type="text/javascript" language="javascript" src="jsme/jsme.nocache.js"></script>  

  
<script>  
 //this function will be called after the JavaScriptApplet code has been loaded.  
    function jsmeOnLoad() {  
        **jsmeApplet** = new JSApplet.JSME("jsme\_container", "380px", "340px");  
   }  
</script>  
</head>  
<body>  
<div id="jsme\_container"></div>  
  
**<button type="button" onclick="alert(jsmeApplet.smiles())">Show SMILES</button>**  
</body>  
</html>

The HTML code above can be tested here.
  
  
The function used to created a new JSME instance can accept a third argument. The purpose of this optional argument is to set options to customize the look and behavior of JSME:

<html>  
  
<head>  
<script type="text/javascript" language="javascript" src="jsme/jsme.nocache.js"></script>  

  
<script>  
 //this function will be called after the JavaScriptApplet code has been loaded.  
    function jsmeOnLoad() {  
        jsmeApplet = new JSApplet.JSME("jsme\_container", "380px", "340px", **{  
            "options" : "oldlook,star"  
            }**);
  
</script>  
</head>  
<body>  
<div id="jsme\_container"></div>  
  
<button type="button" onclick="alert(jsmeApplet.smiles())">Show SMILES</button>  
</body>  
</html>

  
The HTML code above can be tested here.  

## JSME API

The API of JSME includes the API of the java applet JME plus new functions and methods. A summary of the API is presently available as a JavaDoc file.  
  

## JSME options

Several options are available to customize the look and behavior of JSME.

Recognized keywords are :  
  
**xbutton, noxbutton** - show / hide the X button (default is xbutton)  
**rbutton, norbutton** - show / hide the R button (to mark connection of substituent with the main scaffold)  

**atommovebutton, noatommovebutton** - show / hide the atom move button (default is noatommovebutton)  
**hydrogens, nohydrogens** - display / hide hydrogens on heteroatom labels (default is hydrogens)  
**keephs, removehs** - remove hydrogens when reading a molecule with explicit hydrogens (default is keephs). This option can be used to remove hydrogens when pasting a structure with explicit hydrogens comning from e.g. the Pubchem database  
**keephs, removehsc** - remove hydrogens bounded to C when reading a molecule with explicit hydrogens (default is keephs). This option can be used to remove hydrogens when pasting a structure with explicit hydrogens comning from e.g. the Pubchem database  
**query, noquery** - enable / disable query features (default is noquery). In query mode, a SMARTS is generated when a smiles is requested.  
**autoez, noautoez** - automatic generation of SMILES with E,Z stereochemistry (default is autoez)  
**canonize,nocanonize** - SMILES canonicalization and detection of aromaticity supressed (default is canonize)  
**stereo,nostereo** - stereochemistry not considered when creating SMILES (default is stereo)  
**reaction, noreaction** - enable / disable reaction input  
**multipart, nomultipart** - possibility to enter multipart structures (default multipart)  
**addnewpart, noaddnewpart** when reading or pasting a new molecule, add it to the existing molecules or replace them (used only if option multipart is set)  
**polarnitro, nopolarnitro** - prevent automatic conversion of nitro (and similar) groups into nonpolar form (default is nopolarnitro)  
**number / autonumber** - possibility to number (mark) atoms  
**star, nostar** - possibility to highlight an atom (default is nostar). All highlighted atoms will have an atom number equal to 1. The atom numbers are included in the exported SMILES and MOL.  
**depict, nodepict** - the applet will appear without editing buttons, this is used for structure display only (default nodepict)  
**depictaction, nodepictaction** - allow structure editing in depict mode  
**toggle, notoggle** - allow to switch automatically between editing and depict mode (default is notoggle)  
**paste, nopaste** - enabling and disabling the paste action including drop of a MOL/RXN on the applet., can be used together with the depict option to have a read only depiction  
**border, noborder** (used together with the depict option) - displays a border around depicted molecule (default is noborder)  
**newlook, oldlook** - turn on/off the old Java based JME look (default is newlook)  
**exportinchi, noexportinchi** - enable / disable the menu item forInChI export (http://www.inchi-trust.org/)   
**exportinchikey, noexportinchikey** - enable / disable the menu item for InChI key export (http://www.inchi-trust.org/)   
**exportinchiauxinfo, noexportinchiauxinfo** - enable / disable the menu item for InChI auxinfo export (http://www.inchi-trust.org/)   
**searchinchiKey, nosearchinchiKey** - enable / disable the menu item to search InChI key on the web (http://www.inchi-trust.org/)   
**useopenchemlib, nouseopenchemlib** - use the OpenChemLib library (https://github.com/Actelion/openchemlib) for SMILES conversion, 2D coordinates computation and SVG export  
**exportSVG, noexportSVG** - enable / disable the menu item for SVG export (useopenchemlib must be set))   
**useOclidcode, nouseOclidcode** - enable / disable the menu item for OCL export (useopenchemlib must be set))   
**zoom, nozoom** - turn on/off zooming of the molecular drawing area  
**showdraganddropsymbolindepictmode, noshowdraganddropsymbolindepictmode** show or hide the drag and drop symbol (blue triangle) in depict mode  
**atombg** - atom background color - see the demo JSME\_atom\_highlight\_demo.html for an example  
**depictbg** - background color for the depict mode in hex format - no test yet  
  
  
The option of a JMSE instance can be setup during its creation or afterwards using the options() method. See the JSME API section.

## JSME Callbacks

### Introduction

JSME provides a callback mechanism where JavaScript functions are called before or after a specific event has happened in JSME. One of the most useful callback is the "AfterStructureModified" that allows the creation of interactive HTML pages that track any structure change, for instance to compute and display of a 3D structure.
The JavaScript functions are defined in the HTML page and set using the "setCallBack()" method. This method needs two arguments, a callback name and a JavaScript function.

### Example

```
function showSmiles(jsmeEvent) {
	var jsme = jsmeEvent.src;
	var smiles = jsme.smiles();
	alert(smiles);
}

jsmeApplet.setCallBack("AfterStructureModified", showSmiles);
```

### The JSME event variable

Each callback function received one argument: a JSMEevent object. This object contains several fields that may or may be not defined according the event type. The fields of JSMEevent are:

- src: the JSME instance that triggered the event. This variable can be useful if the HTML page contains several instances of JSME.
- action: usually the callback name
- atom: 0 or the atom index affected by the event
- bond: 0 or the bond index affected by the event
- molecule: 0 or the molecule index affected by the event
- argument: null or some meaningful data (e.g. a InchiResult object)

### Predefined callback names

#### BeforePaste AfterPaste AfterStructureModified AtomHighlight BondHighlight AtomClicked BondClicked InchiKeySearch The JSME callback system is work in progress. The InchiResult variable InchiResult objects are generated by the Javascript InChI computation implemented in JSME. The fields of InchiResult are: - inchi - key - auxinfo - warning - error
